# Associations between apparent diffusion coefficient values and histopathological tissue alterations in myopathies

**DOI:** 10.1002/brb3.1809

**Published:** 2020-08-29

**Authors:** Hans‐Jonas Meyer, Ilka Schneider, Alexander Emmer, Malte Kornhuber, Alexey Surov

**Affiliations:** ^1^ Department of Diagnostic and Interventional Radiology University of Leipzig Leipzig Germany; ^2^ Department of Neurology Martin‐Luther‐University Halle‐Wittenberg Halle Germany; ^3^ Department of Diagnostic and Interventional Radiology University of Magdeburg Magdeburg Germany

**Keywords:** MRI, myopathy, myositis

## Abstract

**Objectives:**

Diffusion‐weighted imaging (DWI) can reflect histopathologic changes in muscle disorders. The present study sought to elucidate possible associations between histopathology derived from muscle biopsies and DWI in myositis and other myopathies.

**Methods:**

Nineteen patients (10 women, 52.6%) with a mean age 51.43 ± 19 years were included in this retrospective study. Apparent diffusion coefficients (ADC) were evaluated with a histogram approach of the biopsied muscle. The histopathology analysis included the scoring systems proposed by Tateyama et al., Fanin et al., Allenbach et al. and immunhistochemical stainings for MHC, CD68, CD8, and CD4.

**Results:**

There was a tendency that skewness was lowered with increasing Tateyama score, but it did not reach statistical significance (*p* = .14). No statistical differences for the other scores were identified. There was a tendency that kurtosis was higher in MHC negative stained patient compared to positive patients, but statistically significance was not reached (*p* = .07). ADC histogram parameters did not correlate with CD68 and CD8 positive stained cells. There was a trend for skewness to correlate with the amount of CD4‐positive cells (*r* = .57, *p* = .07).

**Conclusion:**

The present study could not identify statistical significant associations between DWI and histopathology in muscle diseases based upon a small patient sample. Presumably, the investigated histopathology scores are more specific for certain disease aspects, whereas ADC values reflect the whole cellularity of the investigated muscle, which might cause the negative results.

## Graphical abstract

ADC values are not capable to reflect histopathological features in myopathies.

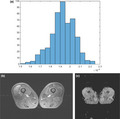

## 1 INTRODUCTION

Myopathies are a heterogeneous group of disorders comprising inflammatory entities with more acute edematous behavior or, for example, dystrophic entities with more degenerative behavior and muscle fiber dysfunction, the first one typically autoimmune myositis and the latter muscle dystrophy (Dalakas, 2011, 2015; Wallace & McNally, 2009). Most of the entities are of unknown etiology (Dalakas, 2011, 2015).

The diagnostic approach in muscle disorders is multimodal consisting of anamnestic features, clinical examination, serological parameters, needle electromyography, and muscle biopsy, used as the gold standard (Dalakas, 2011, 2015; Lundberg, Miller, Tjärnlund, & Bottai, 2016).

Magnetic resonance imaging (MRI) is an established important imaging modality in muscle diseases due to its excellent soft tissue contrast and its possibility of whole body imaging (Bhojwani et al., 2015; Elessawy, Abdelsalam, Abdel Razek, & Tharwat, 2016; Leung, 2017; O'Connell et al., 2002). It can clinically be used to visualize atrophy of affected muscles, muscle edema, and/or myofasciitis (Dalakas, 2015; O'Connell et al., 2002).

Recently, it was shown that diffusion‐weighted imaging (DWI) can be helpful in several muscle disorders comprising tumors, inflammation, and myopathies (Barsotti et al., 2016; Bhojwani et al., 2015; Meyer, Emmer, Kornhuber, & Surov, 2018a, 2018b; Meyer, Höhn, & Surov, 2018; Meyer, Leifels, Hamerla, Höhn, & Surov, 2018; Meyer, Pazaitis, & Surov, 2018; Meyer, Ziemann, et al., 2018; Qi, Olsen, Price, Winston, & Park, 2008; Ran et al., 2016; Surov et al., 2015). DWI reflects random water motion in tissues and is therefore acknowledged to provide deeper insight into tissue microstructure (Surov, Meyer, & Wienke, 2017). It can be quantified by apparent diffusion coefficient (ADC), which correlates inversely with cellularity, amount of extracellular proteins and other tissue factors (Aoyagi et al., 2012; Hauge et al., 2017; Surov et al., 2017).

However, these findings are mainly based upon studies regarding several solid tumors (Surov et al., 2017). Presumably, ADC might also reflect muscle alterations in myopathies and myositis, comprising muscle fiber loss, invasion of inflammatory cells and muscle edema, which has not been investigated previously.

By using histogram analysis, the ADC values of an investigated lesion or structure are issued into a histogram and thereby statistical information regarding heterogeneities can be displayed (Just, 2014). For oncologic imaging, it was shown that this approach can also reflect tumor heterogeneities better and more associations with histopathology could be identified (Just, 2014; Meyer, Emmer, et al., 2018a, 2018b; Meyer, Höhn, et al., 2018; Meyer, Leifels, et al., 2018; Meyer, Pazaitis, et al., 2018; Meyer, Ziemann, et al., 2018).

Therefore, the purpose of the present study was to elucidate, whether ADC parameters investigated with the histogram approach are associated with tissue alterations and/or inflammation in myopathies detected by muscle biopsies.

## 2 MATERIAL AND METHODS

This retrospective study was approved by the institutional ethic committee (Martin‐Luther university of Halle‐Wittenberg) and informed consent was waived.

One hundred and six patients with different muscle disorders were investigated by MRI in our department during the time period 2007 till 2016. Patients were included in this study if they fulfilled the following inclusion criteria:

- muscle disorder confirmed by histopathology;
- patients investigated by MRI including DWI;
- diseased muscles did not show artifacts on DWI, as movement artifacts, signal voids, and susceptibility artifacts;
- available histopathological specimens, preferably obtained from the same region as the MRI within the duration of the same hospitalization to reduce possible bias. No treatment was initiated between biopsy and imaging.

Altogether, 19 patients (10 women, 52.6%) with a mean age 51.4 ± 19.0 years yielded the inclusion criteria.

The diagnoses were as follows: *n* = 5 Polymyositis (26.3%), *n* = 3 Overlap myositis (15.8%), *n* = 2 Dermatomyositis (10.5%), *n* = 2 Limb girdle muscular dystrophy (10.5%), *n* = 2 Spinal muscular atrophy Type II (10.5%), *n* = 2 Neurogenic myopathy (10.5%), *n* = 1 Morbus Pompe (5.3%), *n* = 1 Inclusion body myositis (5.3%), *n* = 1 Myopathy caused by anoctamin 5 mutations (5.3%).

### 2.1 MRI

In every patient, MRI of the thigh and lower leg was performed with a 1.5‐T scanner (Magnetom Vision Sonata Upgrade, Siemens). MRI protocol included turbo spin‐echo (TSE) images, T2‐weighted (T2W) fat‐suppressed short tau inversion recovery (STIR) images, half‐Fourier acquisition single‐shot turbo spin‐echo (HASTE) images, T1‐weighted (T1W) spin‐echo (SE) images prior and after intravenous administration of contrast medium.

DWI was obtained with a SE‐EPI (Single‐shot echo planar imaging) sequence with *b*‐values, 0, and 1,000 s/mm^2^. Motion‐probing gradient pulses were placed in the three orthogonal planes, and isotropic DW imaging was generated by three orthogonal axes. Sequence parameters were as follows: TR/TE: 5,800/68 ms; flip angle: 90°, thickness: 5 mm; matrix size: 128; bandwidth: 2.3 kHz; Imaging Frequency: 63.685; number of averages: 2.

### 2.2 Imaging analysis

**FIGURE 1:**
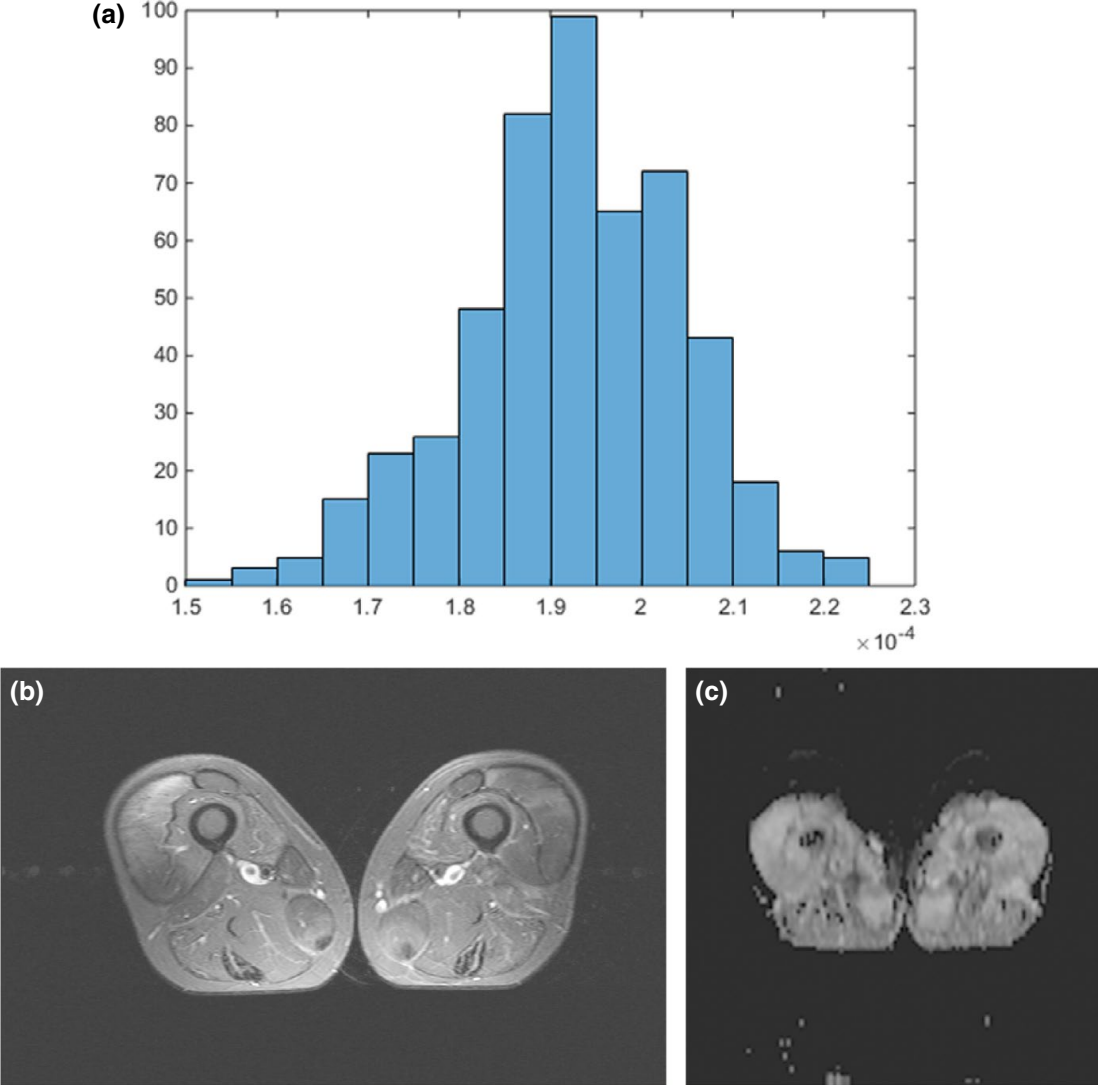
(a) T2‐inversion recovery turbo spin echo‐sequence with fat suppression in axial plane. A hyperintense muscle edema of the quadriceps muscle can be appreciated, on the right side more than on the left. The patient was diagnosed with polymyositis. (b) Corresponding ADC map in axial plane. A slight hyperintense ADC value can be seen in the quadriceps muscle. (c) The resulting histogram of the ADC values. ADC values are reported in (×10^−3^ mm^2^ s^−1^. mean 1.92, min 1.51, max 2.24, p10 1.75, p25 1.85, p75 2.01, p90 2.07, median 1.92, mode 1.90, kurtosis 3.21, skewness −0.26, entropy 3.29. The histopathology score vales are 0 for Allenbach, 1 for Tateyama, and 2 for Fanin

ADC maps were automatically generated by the implemented software and processed offline with custom‐made Matlab‐based application (The Mathworks). Polygonal regions of interest (ROI) were manually drawn on the transferred ADC maps along the contours of the muscle in which the biopsy was obtained. All measures were performed by one radiologist blinded to the histopathology results (A.S., 16 years radiological experience). The following parameters were calculated: ADCmean, ADCmax, ADCmin, ADCmedian, ADCmode, ADC percentiles: 10th, 25th, 75th, and 90th, kurtosis, skewness, and entropy. An explanatory patient is displayed in Figure 1.

### 2.3 Muscle biopsies and histological evaluation

Open muscle biopsies had been taken of the patients as part of the diagnostic work‐up after written consent of all patients. One patient had two biopsies, thus there were 20 biopsies overall. The following muscles were included: Vastus lateralis (*n* = 8, 40%), biceps femoris (*n* = 8, 40%), tibialis anterior (*n* = 2, 10%), adductor magnus (*n* = 1, 5%), and gastrocnemius muscle (*n* = 1, 5%).

For histological analysis, fresh‐frozen sections of muscle biopsies underwent routine histology and immunohistochemistry. For this study, biopsies were retrospectively examined in a blinded manner to the imaging and clinical report by an experienced neurologist well versed in myopathology (I.S.) and different grading was applied for inflammation and dystrophic remodeling.

1. Severity of inflammation in each muscle was graded according to Allenbach et al. 2009, with grading as follows: 1: involvement of a single muscle fiber or <5 muscle fibers; grade 2: a lesion involving 5–30 muscle fibers; grade 3: a lesion involving a muscle fasciculus; and grade 4: diffuse, extensive lesions. When multiple lesions with the same grade were found in a single muscle block, 0.5 points were added to the grade.
2. We used the scoring system that was published by Tateyama et al., 2015 with separate grading of inflammation and necrosis and regeneration. The extent of mononuclear cell infiltration (mononuclear cell infiltration score) was graded as follows: grade 0: none or slight; grade 1: one focus of mononuclear cell infiltration; grade 2: more than one focus of mononuclear cell infiltration; and grade 3: diffuse mononuclear cell infiltration. Muscle fiber necrosis and regeneration (necrosis/regeneration score) were graded as follows: grade 0: none; grade 1:1% or less of muscle fibers showing necrosis or regeneration; grade 2: more than 1% and no more than 10% of muscle fibers showing necrosis or regeneration; and grade 3: more than 10% of muscle fibers showing necrosis or regeneration. The total histological scores were calculated for each patient by adding the mononuclear cell infiltration and the necrosis/regeneration scores.

Additionally, we used a 4‐point histopathological scale based on the dystrophic changes (histology severity score HHS) as was introduced by Fanin et al., 2007. HSS grade was as follows: grade 1: mild (slight increase in fiber size variability, absent or mild regeneration and degeneration, absent or mild fibrosis); grade 2 moderate (moderate increase in variability of fiber size, variable degeneration, and regeneration, moderate or severe fibrosis); grade 3 severe (marked increase in variability of fiber size, variable degeneration and regeneration, moderate, or severe fibrosis; and grade 4 advanced (huge increase in variability of fiber size, variable degeneration, and regeneration, severe fibrosis).

Immunohistochemistry included staining for MHC‐I, CD68, CD8, and CD4.

### 2.4 Statistical analysis

Statistical analysis and graphics creation were performed using GraphPad Prism (GraphPad Software). Collected data were evaluated by means of descriptive statistics (absolute and relative frequencies). Spearman's correlation coefficient (*r*) was used to analyze associations between investigated parameters. ADC and clinical subgroups were analyzed by Mann–Whitney test. In all instances, *p* values < .05 were taken to indicate statistical significance.

## 3 RESULTS

The mean values of the ADC parameters are summarized in Table 1.

**TABLE 1:** Overview of the ADC parameters

ADC parameter (×10^−3^ mm^2^ s^−1^)	Mean ± *SD*	Range
Mean	1.78 ± 0.28	1.45–2.39
Min	1.19 ± 0.26	0.61–1.59
Max	2.38 ± 0.45	1.87–3.44
P10	1.52 ± 0.26	0.95–2.05
P25	1.64 ± 0.27	1.08–2.2
P75	1.91 ± 0.30	1.4–2.54
P90	2.03 ± 0.33	1.53–2.77
Median	1.78 ± 0.28	1.45–2.37
Mode	1.76 ± 0.31	1.25–2.41
Kurtosis	3.34 ± 1.68	1.86–10
Skewness	0.04 ± 0.47	−0.82–1.23
Entropy	3.11 ± 0.46	2.24–4.15

For the dystrophy score HSS (Fanin) one patient had four points (5.3%), five had two points (26.3%) and 13 patients had one point (68.4%). There were no significant differences regarding ADC parameters between the group with one point to the group comprising more points.

For the inflammation scores, Allenbach 4 was identified in one patient (5.3%), 1.5 points in three patients (15.8%), one point in three patients (15.8%) and zero points in 12 patients (63.2%).

Tateyama 6 was identified in one patient (5.3%), four points in two patients (10.6%), three points in one patient (5.3%), two points in two patients (10.6%), and one point in four patients (21.1%), zero points were identified in eight patients (42.1%).

**FIGURE 2:**
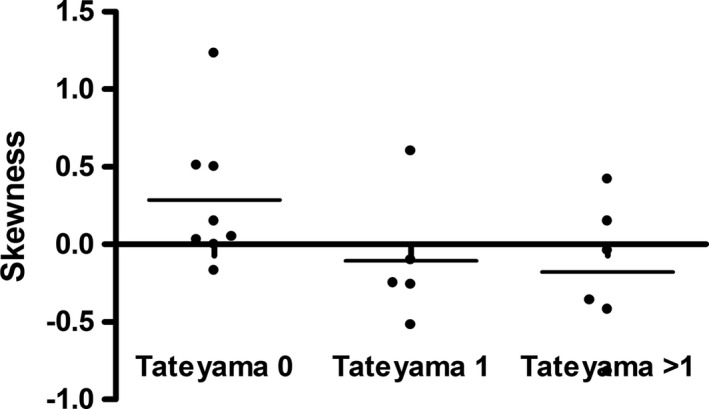
Scatter plot displays ADC skewness in dependence of Tateyama score. There was a statistical trend that skewness was lowered with increasing Tateyama score (*p* = .14)

There was a tendency that skewness was lowered with increasing Tateyama score, but it did not reach statistical significance (*p* = .14, Figure 2).

### 3.1 Immunohistochemical analysis

Immunohistochemical analysis was available for 11 patients.

**FIGURE 3:**
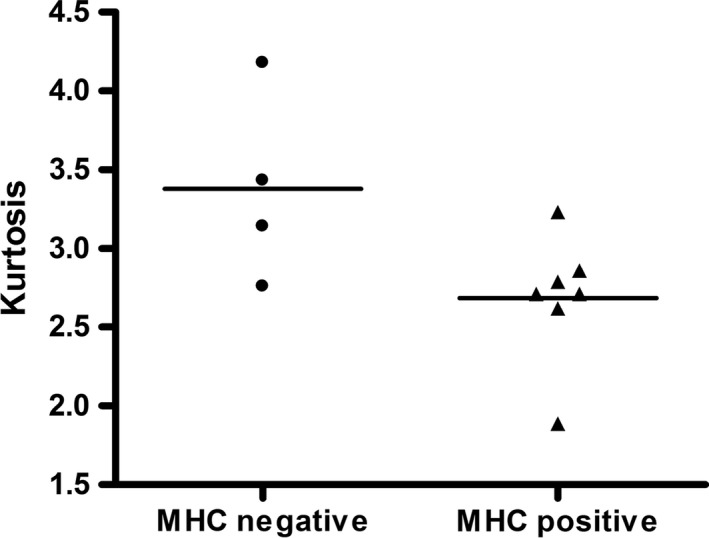
Scatter plot displays comparison between MHC stained positive and MHC negative patient in regard of kurtosis. There was a trend that kurtosis was higher in MHC negative patient, yet statistical significance was not reached (*p* = .07)

Regarding MHC staining, one patient had three points (9.1%), one patient 2.5 points (9.1%), one patient had two points (9.1%), four patients had one point (36.4%) and four patients (36.4%) had no visible staining. There was a tendency that kurtosis is higher in MHC negative stained patients compared to positive stained, but statistically significance was not reached (*p* = .07, Figure 3).

There were no correlations with CD68 and CD8 positive stained cells.

**FIGURE 4:**
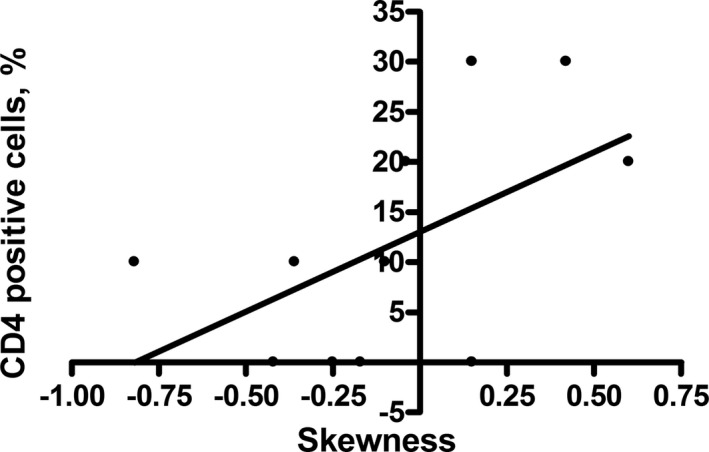
Correlation analysis between CD4 stained positive cells and skewness. Spearman's correlation coefficient is *r* = .57, *p* = .07

There was a trend for skewness to correlate with the amount of CD4‐positive cells (*r* = .57, *p* = .07, Figure 4).

## 4 DISCUSSION

The present study found some correlations between intramuscular alteration and inflammation parameters and DWI in muscle diseases. To the best of our knowledge, this is the first investigation analyzing complex histopathological findings and DWI in muscle disorders.

Previously, it has been extensively shown that ADC values derived from DWI can reflect microstructure of tissues 2018a, 2018b; Meyer, Höhn, et al., 2018; Meyer, Leifels, et al., 2018; Meyer, Pazaitis, et al., 2018; Surov et al., 2017). Hereby, the principle is that protons move faster in the extracellular space than intracellularly. Therefore, movement in this extracellular space is the main factor of the signal intensity obtained with DWI. Muscle tissue is a densely packed tissue with linear located structure involving long fibers, and myofilaments (Oudeman et al., 2016). In pathologic tissue alterations, for example in tumors, there is higher cellularity caused by proliferating cells, which reduces the extracellular space and consecutively reduces the proton movement overall, resulting in a lower ADC value in tumors. Moreover, it was shown that other tissue features, such as extracellular matrix proteins are also associated with ADC values (Aoyagi et al., 2012; Hauge et al., 2017). Additionally, there is an influence of perfusion to the DWI signal intensity (De Luca, Bertoldo, & Froeling, 2017; Le Bihan et al., 1988).

Therefore, DWI has been investigated in several muscle disorders, comprising neoplastic lesions, inflammatory lesions, myositis, and dystrophic disorders with very promising results to be a valuable addition to morphological imaging (Barsotti et al., 2016; Bhojwani et al., 2015; Meyer, Emmer, et al., 2018a, 2018b; Meyer, Höhn, et al., 2018; Meyer, Leifels, et al., 2018; Meyer, Pazaitis, et al., 2018; Meyer, Ziemann, et al., 2018; Qi et al., 2008; Ran et al., 2016; Surov et al., 2015). For example, it may aid in differentiation between disease entities or even help in treatment evaluation (Qi et al., 2008).

Presumably, these findings are directly influenced by tissue alterations in muscle tissues. These are complex and can change during the time course of the disease. So, in the acute phase, inflammation and edema are present, whereas in the chronic phase dystrophy and degeneration of the muscle fibers outweigh (Dalakas, 2011, 2015).

In detail, T cells invade the endomysium (Dalakas, 2011). Secondly, due to the inflammation reaction muscle fibers degrade by apoptosis and necrosis (Dalakas, 2015). Then, extracellular edema is detectable by morphological MRI (O'Connell et al., 2002). Presumably, the diffusion might be initially restricted due to increasing cellularity by the inflammatory cells. Later, the diffusion might be elevated due to necrosis of the muscle fibers with more free diffusion space for water molecules (Dalakas, 2011, 2015). However, these are only hypothesis and have not been systematically investigated yet.

For myositis, one study identified a lower ADC value for diseased muscles compared to nondiseased control muscles (Ran et al., 2016), whereas two other studies identified a higher ADC value in affected muscles (Meyer, Ziemann, et al., 2018; Qi et al., 2008). The findings of the first study might be explained by increased cellularity induced by invading inflammation cells and thus a lowered ADC value. Moreover, the cytotoxic induced reaction by CD8‐positive cells might not yet be present, which would lead to elevated diffusion due to increased extracellular space.

The induced cytotoxic reaction and the inflammatory induced edema might be related to the results of the two studies identifying higher ADC values in affected muscles (Meyer, Emmer, et al., 2018a, 2018b; Meyer, Ziemann, et al., 2018; Qi et al., 2008). As a concern, these mentioned studies did not have histopathology confirmation to elucidate the underlying causes of ADC value alteration.

A recent study could show that MRI characteristics, especially T2‐weighted sequence, muscle pathology, and expression of DUX4 target genes in a patient sample of facioscapulohumeral muscular dystrophy (Wang et al., 2019). So, MRI findings can be used to identify muscles with elevated disease activity in this muscle disorder.

The importance of T2‐weighted derived parameters was also highlighted in a recent study, which could show associations between entropy derived from T2‐weighted images and CD20, CD4, and CD8 cells as well as with MHC‐I expression (Meyer, Schneider, Emmer, Kornhuber, & Surov, 2020). Presumably, T2‐weighted imaging is more important in muscle disorders than the DWI.

In another study it was identified that ADC values strongly correlated with electromyography findings in myositis, indicating the capability of ADC values to reflect functional aspects of muscle tissues (Meyer, Emmer, et al., 2018a, 2018b).

Interestingly, the present study could identify a tendency for ADCmode and skewness to correlate with the amount of CD4 stained cells but not with CD8 stained cells. Presumably, statistical significance was not reached due to our small patient sample. CD4 is a glycoprotein, which is expressed on T‐helper cells, monocytes and macrophages. Due to interactions with CD8‐positive killer cells, they mediate cytotoxic immune response, which is the predominantly pathway in myositis. In polymyositis, CD8‐positive lymphocytes are located in the endomysium surrounding healthy muscle fibers. The cytotoxic reaction is with MHC‐1 class antigen (Dalakas, 2015). We could identify a statistically trend between kurtosis and the staining expression of MHC1‐protein.

A concern might be that the MRI was performed later in the clinical manifestation in which the muscle degeneration has a higher impact on the ADC value than the cell component resulting in an overall higher ADC value. In a recent study investigating oropharyngeal cancer, ADC values correlated negatively with CD3‐positive cells, comprising the whole amount of T cells within the tumor (Swartz et al., 2018). This can be assessed as first evidence that ADC values might therefore be sensitive enough to reflect microenvironmental characteristics of tumors and not only the whole amount of cell density (Divine et al., 2016; Swartz et al., 2018).

The present study could only identify a statistical trend for increasing ADC skewness and concordantly increasing Tateyama score and no associations with the other scores. This might be related to the fact that these scores are more related to specific muscle alterations, whereas ADC values reflect the whole amount of cellularity in tissues (Surov et al., 2017).

A strength of the present study is the measurement of the ADC values with a histogram analysis, which has been shown to be superior than the conventional measurement (Meyer, Emmer, et al., 2018a, 2018b; Meyer, Höhn, et al., 2018; Meyer, Leifels, et al., 2018; Meyer, Pazaitis, et al., 2018; Meyer, Ziemann, et al., 2018). With this approach statistical information regarding heterogeneity of tissues can be obtained). Regarding myositis this technique has been used in a previous study and only kurtosis derived from ADC values was associated with C‐reactive protein serum level in this entity (Meyer, Emmer, et al., 2018a, 2018b). Presumably, with this measurement associations with histopathology in muscle disorders might also be better reflected. For oncologic imaging, it has been widely shown that the histogram approach is capable to reflect tumor heterogeneity in several tumor entities and that different histogram parameters also reflect different aspects of tumor microstructure (Meyer, Höhn, et al., 2018; Meyer, Leifels, et al., 2018; Meyer, Pazaitis, et al., 2018; 2018; Meyer, Hamerla, Höhn, & Surov, 2019; Surov, Meyer, Winter, Richter, & Hoehn, 2018).

Several limitations of this study have to be addressed. Firstly, it has a retrospective design with inherent possible bias. However, imaging and histopathology were performed independently and blinded to each other to reduce possible bias. Secondly, our patient sample is relatively small, which other similar studies also suffer from. As another concern the patient sample comprises heterogeneous disease entities. Thirdly, albeit the same muscle was investigated with the MRI there might be still some local incongruencies between biopsy and MRI, which might have an impact on the results. Lastly, we obtained DWI with only two *b*‐values and, therefore, could not calculate other DWI parameters such as perfusion fraction (*f*) or true Diffusion (*D*), which might have stronger associations with the investigated histopathology (Le Bihan et al., 1988).

Clearly, studies with prospective design and a larger patient sample are needed to further characterize the complex interactions between histopathology and DWI in muscle disorders.

## 5 CONCLUSION

The present study could not identify statistically significant associations between DWI and histopathology in muscle diseases based upon a small patient sample. Presumably, the investigated histopathology scores are more specific for certain disease aspects, whereas ADC values reflect the whole cellularity of the investigated muscle, which might cause the negative results.

## CONFLICT OF INTEREST

None to declare.

## AUTHOR CONTRIBUTION

AS and HJM designed and conceptualized the study. HJM analyzed the data and drafted the manuscript. HJM, AM, IS, MK mainly collected the data. AS and MK revised the manuscript for intellectual content.

### Peer Review

The peer review history for this article is available at https://publons.com/publon/10.1002/brb3.1809.

## Data Availability

Qualified researchers may request access to patient‐level data and related study documents including the clinical study report, study protocol with any amendments, blank case report form, statistical analysis plan, and dataset specifications. Patient‐level data will be anonymized and study documents will be redacted to protect the privacy of trial participants.
